# Efficacy, safety and tolerability of field treatment of actinic keratosis with ingenol mebutate 0.015 % gel: a single center case series

**DOI:** 10.1186/s40064-016-2290-6

**Published:** 2016-05-14

**Authors:** Ivan Bobyr, Anna Campanati, Veronica Consales, Katia Giuliodori, Alessandro Scalise, Annamaria Offidani

**Affiliations:** Dermatological Unit, Department of Clinical and Molecular Sciences, Polytechnic Marche University, Ancona, Italy; Department of Plastic and Reconstructive Surgery, Polytechnic Marche University, Ancona, Italy

**Keywords:** Actinic keratosis, Ingenol mebutate, Field cancerization

## Abstract

**Introduction:**

Actinic keratosis (AK) is a cutaneous intraepithelial neoplasm appearing within areas referred as ‘fields of cancerization’. AK can progress to invasive squamous cell carcinoma. Treatments that target both clinically visible and subclinical AKs in cancerization fields are able to reduce the risk of malignant progression. Ingenol mebutate gel is a new effective topical therapy for AK, used once daily for 2 or 3 days depending on the location of lesions.

**Case description:**

Three elderly patients with multiple non-hypertrophic AKs within a contiguous 25-cm^2^ treatment area on the face or scalp were treated with ingenol mebutate 0.015 % gel once daily for three consecutive days and followed up over a period of 57 days.

**Discussion and Evaluation:**

Although individual local responses to treatment varied, all patients had total clearance of AK lesions without any sign of recurrence. In addition, all patients said that they were satisfied with the effectiveness of ingenol mebutate treatment and the aesthetic outcome, and would be prepared to use this agent again to treat AK in the future, if necessary.

**Conclusions:**

These three cases demonstrate that ingenol mebutate 0.015 % gel is effective and well tolerated in a clinical setting, with effective clearance of AK lesions present on the face and scalp, and good patient acceptability.

## Background

Actinic keratosis (AK) is a cutaneous intraepithelial neoplasm [early-stage squamous cell carcinoma (SCC)] that is relatively common in elderly individuals with sun-damaged skin. AK is the most common cutaneous lesion encountered in clinical practice and its incidence is increasing worldwide (Ortonne 2002). There are a number of reasons for this, including the thinning of the ozone layer (which results in more ultraviolet radiation reaching earth) and lifestyle factors (such as the desire to achieve tanned skin) (Chetty et al. 2015).

Characteristic features of AK include keratotic macules, papules, or plaques with superficial scales on a red background, which are classified as hypertrophic, bowenoid, atrophic, acantholytic, epidermolytic, lichenoid and/or pigmented based on histological features. AK lesions may be sore or itchy but many patients experience no symptoms at all (Moy 2000).

A dermoscopic classification, useful for diagnosis of pigmented and non-pigmented skin lesions, has recently been proposed that recognizes 3 different grades of classical non-pigmented AK (Grade 1, 2, or 3), plus pigmented and lichenoid AK (Zalaudek and Argenziano 2015). Grade 1 refers to lesions that are slightly palpable and better felt than seen, Grade 2 to many visible, small, moderately thick or a few large, thick, rough scaly lesions, and Grade 3 to many thick, hyperkeratotic lesions that are clearly visible and palpable with well-defined borders. Furthermore, AKs show increased vascularization (neo-angiogenesis) and have higher capillary densities than surrounding normal tissue, both of which become more severe and evident in invasive skin lesions (Cantisani et al. 2015).

If left untreated, some AK lesions have the potential to progress to invasive SCC (iSCC); data suggest that 72–82.4 % of identified SCCs arise contiguously with, or within and/or in close proximity to, AKs (Czarnecki et al. 2002). While there is no way of predicting exactly which individual AK lesions will progress into iSCC, the risk of such progression has been estimated to be 0.6 % over 1 year and 2.57 % over 4 years (Werner et al. 2013). In the absence of reliable ways to predict progression of AK lesions to iSCC, treatment of the ‘field of cancerization’ is an important aspect of AK treatment. This term was originally used by Slaughter et al. in 1953 to refer to histologically abnormal epithelium adjacent to tumor tissue and was designed to explain the occurrence of multiple primary tumors as well as locally recurrent cancer (Kaufman 2010; Slaughter et al. 1953).

There are a number of options available for the management of AKs. The specific choice of approach depends on a number of factors, including lesion distribution, number and thickness, as well as previous treatments and episodes of recurrence. Options directly targeting the lesion include cryotherapy, laser therapy, curettage and dermabrasion; field-directed therapies are imiquimod, photodynamic therapy (PDT) and ingenol mebutate (Goldenberg and Perl 2014). Field-directed therapies have the ability to target both clinically visible and subclinical AKs in a cancerization field, meaning that they have a greater potential to reduce the risk of malignant progression.

Ingenol mebutate is a diterpene ester derived from the sap of the Euphorbia peplus (petty spurge) plant. Topical application is associated with both chemo-ablative and immunomodulatory effects (Dodds et al. 2014). This combination of activity results in preferential cell death in transformed keratinocytes and an inflammatory reaction that kills any remaining cancerous cells (Martin and Swanson 2013). However, the precise mechanism of action of ingenol mebutate is still the subject of research. A recent study suggested that ingenol mebutate-induced cell death is mediated through the PKCδ/MEK/ERK pathway, and that downstream induction of IL1R2 and IL13RA2 expression reduce the viability of treated cells (Freiberger et al. 2015).

This series of cases provides treatment and follow-up data on the efficacy, safety and tolerability of ingenol mebutate 0.015 % gel for the treatment of AKs on the face or scalp.

## Case presentation

We present details for 3 elderly patients with multiple (>6) lesions non-hypertrophic AK lesions within a contiguous 25-cm^2^ treatment area on the face or scalp. All patients were treated with ingenol mebutate 0.015 % gel once daily for three consecutive days, then assessed after 4 days for evaluation of local skin response (LSR) and recording of adverse events. A third assessment was performed after 15 days to evaluate the LSR, treatment efficacy [defined as total clearance (complete disappearance of all the lesions) or partial clearance (75 % reduction in the number of lesions) in the treated area], and adverse events. The final evaluation at 57 days assessed the LSR and definitive treatment results (total or partial clearance), adverse events (appearance of scars, hyperpigmentation, hypopigmentation), patient satisfaction [rated on a visual analogue scale (VAS) from 0 (extremely dissatisfied) to 10 (extremely satisfied)], and any requirement for retreatment.

All patients were given verbal and written information about the treatment procedures, LSR and possible treatment-related side effects. Written informed consent was obtained from all participants in accordance with the latest revision of the Declaration of Helsinki (2009/58). Patients provided consent to the publication of their case details and the related images.

### Case 1

**Fig. 1:**
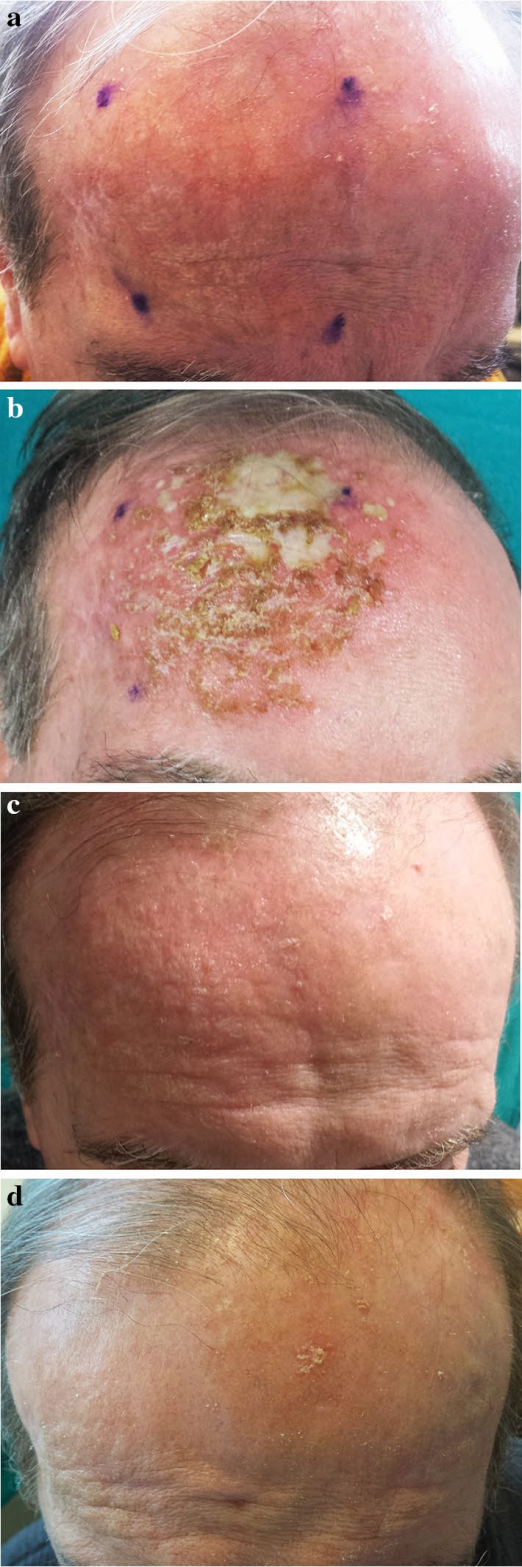
**a** Clinical presentation at base-line; **b** LSR on day 4; **c** 15 days of follow-up; **d** 57 days of follow-up

A 78-year-old man presented to the clinic with a 3-year history of multiple erythematous, scaly macules and papules on a red base located on the frontal region. The patient also reported a history of relapsing, remitting lesions a few months after multiple cryotherapy sessions. Physical examination showed multiple rough, dry, erythematous, scaly and raised bumps and patches that were 0.5–1.0 cm in size (Fig. 1a). Dermoscopic examination revealed the presence of hair follicle openings filled with yellowish keratotic plugs and surrounded by a white halo with a targetoid-like appearance on a red background (“strawberry pattern”). The patient was treated with ingenol mebutate 0.015 % gel on the right frontal 25-cm^2^ treatment area for 3 consecutive days. LSR on day 4 was severe (Fig. 1b), with edema, erythema, confluent pustules, erosions, severe crusts and periorbital edema, but no additional treatment was required and the patient’s adherence to treatment was not affected. The LSR had resolved completely after 15 days without scars (Campanati et al. 2010) or skin color change, and healthy skin with complete AK clearance was achieved (Fig. 1c). Most of the dermoscopic features of AKs disappeared after ingenol mebutate treatment. Total clearance of AK lesions was observed after 57 days of follow-up without any sign of recurrence (Fig. 1d). The patient reported that he was satisfied (VAS score = 9) with the effectiveness of treatment and the aesthetic outcome, and said he would be prepared to use the same treatment again in future, if necessary.

### Case 2

**Fig. 2:**
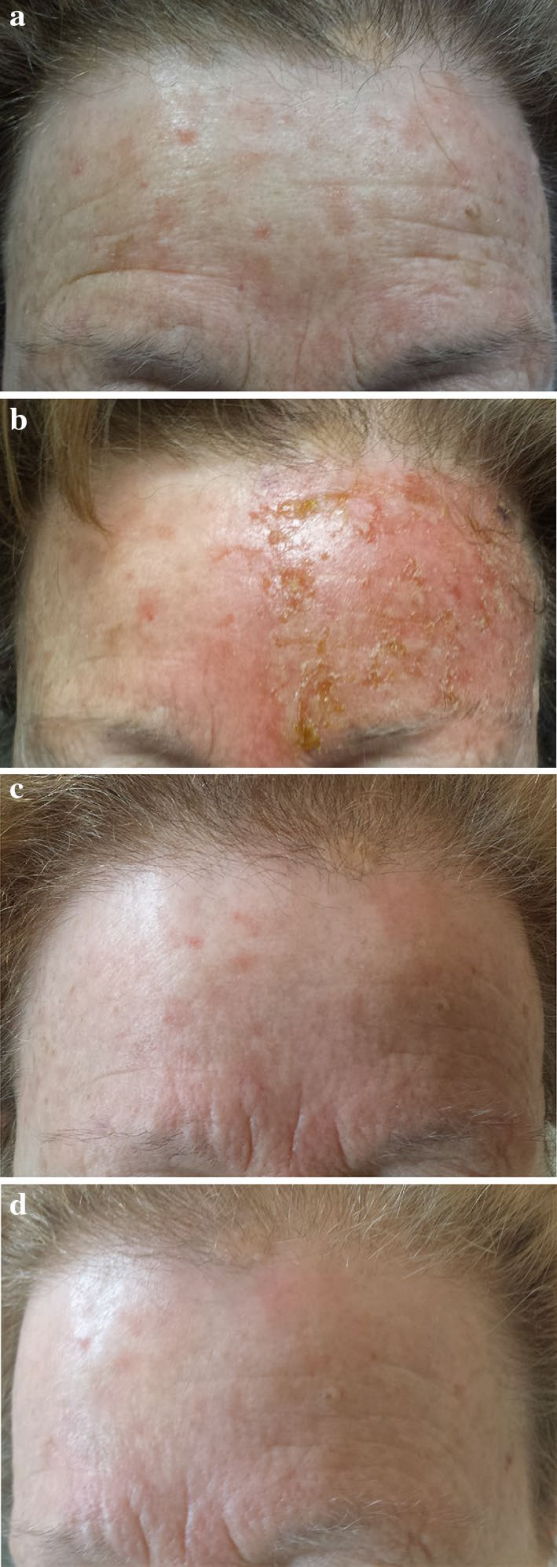
**a** Clinical presentation at base-line; **b** LSR on day 4; **c** 15 days of follow-up; **d** 57 days of follow-up

An elderly woman (age 73 years) with a 4-year history of multiple discrete non-hypertrophic and pigmented AKs within the frontal region presented for treatment. Physical examination showed multiple erythematous macules, scaly and raised bumps, and brown patches (0.3–1.2 cm in size) on sun-damaged skin (Fig. 2a). Annular–granular structures characterized by the coalescence of small, grey to brown dots and globules around hair follicles and typical brown pseudo-network were evident on dermoscopy. Treatment with ingenol mebutate 0.015 % gel was prescribed, which the patient applied to the left frontal 25-cm2 treatment area for 3 consecutive days. LSR on day 4 was moderate (Fig. 2b), with edema, erythema, some pustules, mild erosion and a few crusts. The patient did not need any additional treatment and there was complete resolution of LSR after 15 days without sequelae, and skin photorejuvenation with complete AK lesion clearance was observed (Fig. 2c). Most of the initial dermoscopic features of AK lesions had disappeared on reassessment after treatment. At 57 days’ follow-up, there was total clearance of AK lesions without signs of recurrence (Fig. 2d). The patient was very satisfied with the clinical and aesthetic outcomes of treatment (VAS score = 10) and stated her willingness to undergo another ingenol mebutate treatment course if necessary in the future.

### Case 3

**Fig. 3:**
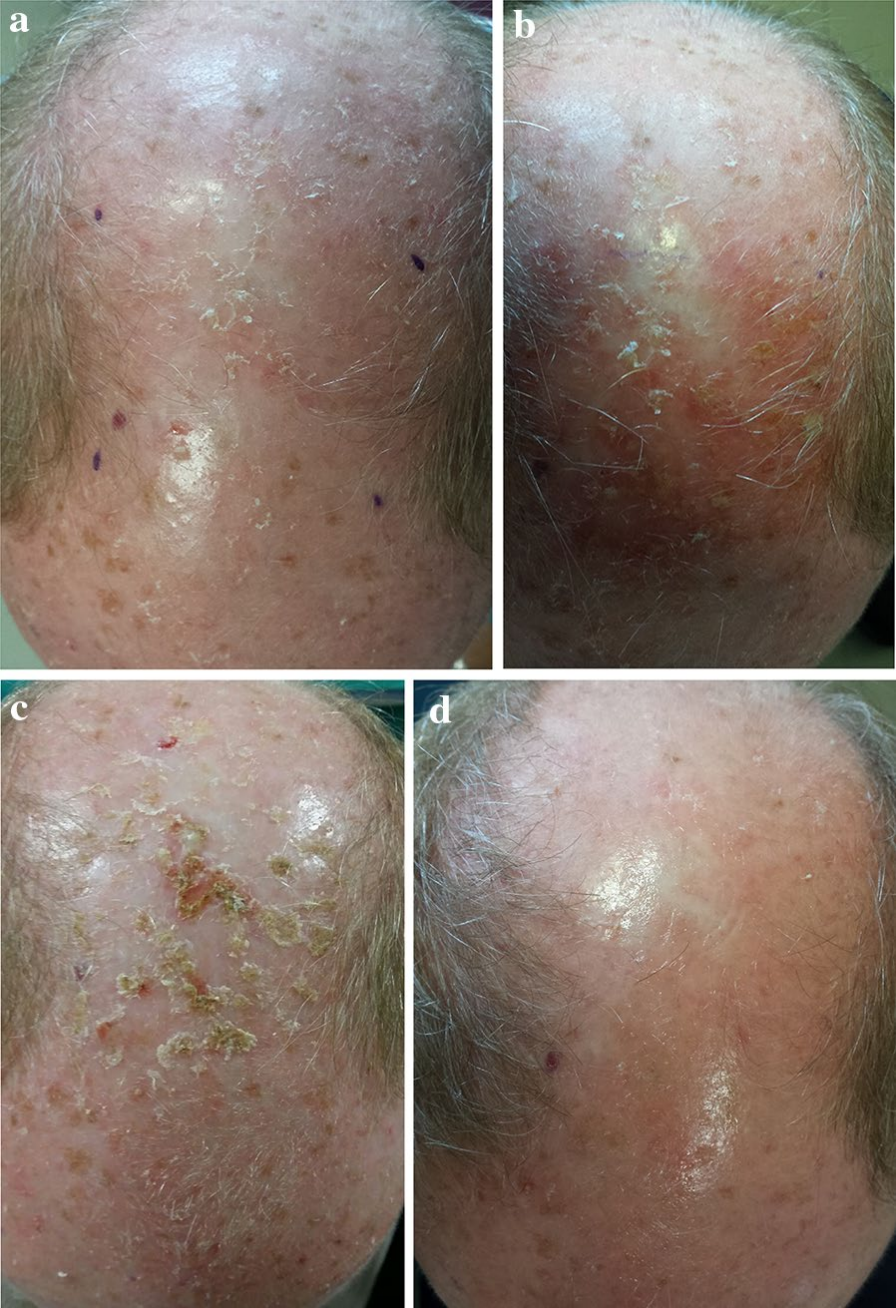
**a** Clinical presentation at base-line; **b** LSR on day 4; **c** 15 days of follow-up; **d** 57 days of follow-up

A 62-year-old male with fair skin presented with multiple erythematous, scaly macules and papules on the fronto-parietal scalp area that had been present for 2 years. He did not report any soreness or itch. Physical examination showed multiple, moderately thick erythematous scaly lesions and other signs of solar damage, such as solar elastosis (Fig. 3a). Dermoscopic examination revealed the presence of unfocussed large caliber vessels located between the hair follicles and arranged in a reddish pseudo-network with discrete white scale. Treatment with ingenol mebutate 0.015 % gel for 3 consecutive days was prescribed and the patient was instructed to apply this to a 25-cm^2^ treatment area on the vertex for 3 consecutive days. LSR on day 4 was mild (Fig. 3b), with application-site pruritus, very mild erythema, few pustules, some scales and crusts. The patient did not experience any LSR-related symptoms and did not require any additional treatment. Crusts persisted for the following 2 weeks (Fig. 3c), but all had disappeared within another week after that. Complete resolution of LSR was evident after 22 days, without scars, hyperpigmentation, hypopigmentation, and complete AK lesion clearance was obtained. Most of the dermoscopic features of AK disappeared after ingenol mebutate treatment. There was total clearance of AK lesions after 57 days and no signs of recurrence (Fig. 3d). This patient was also satisfied with the clinical results associated with ingenol mebutato (VAS score = 10) and said he would use the treatment again in the future if he needed to.

## Discussion

There are a number of field therapies available for the treatment of AK, including imiquimod 5 %, 5-fluorouracil (5-FU), diclofenac 5 %, hyaluronic acid 2.5 %, and PDT (Dodds et al. 2014). The usefulness of some agents is limited by a need for prolonged use and the occurrence of associated inflammatory effects, which decrease the tolerability and acceptability of treatment, which in turn could reduce patient adherence (Shergill et al. 2013). On that basis, the ideal treatment for AK in the clinical setting would be one that is easy and convenient to use, provides good and sustained clearance of AK lesions, reduces the risk of malignant transformation, and is well tolerated (Garbe et al. 2016).

Data from a number of clinical studies suggest that ingenol mebutate is an effective and well tolerated field-directed treatment for AK (Garbe et al. 2016; Lebwohl et al. 2012, 2013). Furthermore, the findings of a meta-analysis comparing different treatments for AK of the face or scalp showed that clearance rates with ingenol mebutate were similar to those of imiquimod 5 % and 5-FU for 4 weeks, and were superior to those of diclofenac, imiquimod 3.75 % and cryotherapy (Vegter and Tolley 2014). LSRs (including erythema, edema, scaling, crusting, swelling, vesiculation and pustulation, erosion or superficial ulceration at the application site) are the most common side effects of ingenol mebutate, but usually resolve spontaneously within 2 weeks. Transient application-site pain and itch are the most commonly reported ingenol mebutate-induced adverse events (AEs) (Rosen et al. 2014). Data from a recent prospective, randomized study indicated that although the peak intensity of LSR was similar for ingenol mebutate 0.015 % gel and 5-FU 5 %, the area under the curve for LSRs and pain was significantly lower with ingenol mebutate (Samorano et al. 2015).

The outcomes in our three patients are consistent with currently available data for ingenol mebutate in AK, which show sustained lesion reduction, clinically-relevant clearance and reduced numbers of lesions in the treated field. In addition, our use of ingenol mebutate in clinical practice confirmed that this agent is well tolerated, with a safety profile comparable to that in previous studies. The LSRs experienced by our treated patients were mild to moderate and all resolved without sequelae within 2 weeks of treatment initiation. None of the patients needed any additional treatment and LSRs did not influence their adherence to treatment. In addition, all three patients were satisfied with the clinical efficacy and aesthetic outcome of ingenol mebutate treatment and said they would be willing to use the drug again in the future if necessary.

## Conclusions

These three cases demonstrate the efficacious and safe use of ingenol mebutate 0.015 % gel in a clinical setting, with effective clearance of AK lesions present on the face and scalp. Our experiences adds to data showing that ingenol mebutate can be used for field treatment of AK, demonstrating an acceptable safety profile and a high sustained clearance rate. The benefits of ingenol mebutate in this setting include the short duration of treatment, ease of self-application, transient, self-limiting adverse effects, and an improvement in skin quality.

## Abbreviations

AE: adverse event
AK: actinic keratosis
LSR: local skin response
PDT: photodynamic therapy
SCC: squamous cell carcinoma
VAS: visual analogue scale
